# From Blood to the Brain: Can Systemically Transplanted Mesenchymal Stem Cells Cross the Blood-Brain Barrier?

**DOI:** 10.1155/2013/435093

**Published:** 2013-08-12

**Authors:** Linan Liu, Mark A. Eckert, Hamidreza Riazifar, Dong-Ku Kang, Dritan Agalliu, Weian Zhao

**Affiliations:** ^1^Department of Pharmaceutical Sciences, Sue and Bill Gross Stem Cell Research Center and Chao Family Comprehensive Cancer Center, University of California, Irvine, 845 Health Sciences Road, Irvine, CA 92697, USA; ^2^Department of Biomedical Engineering and Edwards Lifesciences Center for Advanced Cardiovascular Technology, University of California, Irvine, 845 Health Sciences Road, Irvine, CA 92697, USA; ^3^Department of Developmental & Cell Biology, University of California, Irvine, 4236 McGaugh Hall, Irvine, CA 92697, USA

## Abstract

Systemically infused mesenchymal stem cells (MSCs) are emerging therapeutics for treating stroke, acute injuries, and inflammatory diseases of the central nervous system (CNS), as well as brain tumors due to their regenerative capacity and ability to secrete trophic, immune modulatory, or other engineered therapeutic factors. It is hypothesized that transplanted MSCs home to and engraft at ischemic and injured sites in the brain in order to exert their therapeutic effects. However, whether MSCs possess the ability to migrate across the blood-brain barrier (BBB) that separates the blood from the brain remains unresolved. This review analyzes recent advances in this area in an attempt to elucidate whether systemically infused MSCs are able to actively transmigrate across the CNS endothelium, particularly under conditions of injury or stroke. Understanding the fate of transplanted MSCs and their CNS trafficking mechanisms will facilitate the development of more effective stem-cell-based therapeutics and drug delivery systems to treat neurological diseases and brain tumors.

## 1. Introduction

Despite enormous advances in our understanding of the molecular and cellular basis of neurological diseases, therapies that lead to sustained improvement or resolution of symptoms have remained elusive. Regenerative therapeutics, that encompass embryonic, neural, and adult stem cell therapies, possess great potential to reverse neuronal damage associated with CNS diseases such as stroke, multiple sclerosis (MS), Parkinson's disease (PD), and Alzheimer's disease (AD) [1]. Mesenchymal stem cells (MSCs) are an especially attractive therapeutic agent due to their ease of isolation, established safety, and potential to target multiple pathways involved in neuronal regeneration. MSCs are connective tissue progenitors that can be readily isolated from multiple tissues including bone marrow and adipose tissue [2–4]. While being initially used for treatment of connective tissue disorders due to their potential to differentiate into bone, cartilage, and fat cells, the discovery that MSCs can secrete cytokines and growth factors with antiapoptotic, proangiogenic, neuroprotective, and immune-modulatory properties has sparked broad clinical interest [2–4]. In fact, MSCs are the world's first manufactured stem cell product (i.e., Osiris's Prochymal) approved in Canada to treat graft-versus-host disease (GvHD) [5]. MSCs are currently being tested for treating some neurological diseases in multiple ongoing clinical trials, although their exact therapeutic mechanisms *in vivo* remain largely unknown (i.e., immunomodulation versus secretion of trophic factors that promote tissue regeneration and vascularization) [1, 6, 7]. Furthermore, there is great interest in using MSCs as vehicles to deliver antitumor therapeutics (e.g., tumor necrosis factor-related apoptosis-inducing ligand (TRAIL), interferon-*β*, and oncolytic viruses) for brain tumor treatment [8–10]. 

Given the large number of ongoing clinical trials that use systemic infusion (i.e., intravenously (IV) and intra-arterially (IA)) of MSCs expanded *in vitro *[2, 3], a procedure that is minimally invasive and convenient, it is critical to understand if transplanted MSCs can home to and engraft at ischemic and injured sites in the brain to exert their therapeutic effects. Currently, it is unclear whether systemically infused MSCs can actively migrate across the blood-brain barrier (BBB) that separates the blood and brain. This review attempts to synthesize the recent literature on MSC brain tropism, MSC/BBB interactions, and the underlying molecular mechanisms. We will first briefly introduce how leukocytes and tumor cells transmigrate across the BBB, especially under pathological conditions, to provide a mechanistic framework for the subsequent discussion on MSC homing. We will then concentrate on *in vivo* and *in vitro* studies that address whether MSCs actively interact with and transmigrate across the BBB, molecular mechanisms involved in the tropism of MSCs to the injured brain, interactions with the BBB, and biological/therapeutic implications to using MSCs as trophic vehicles for CNS drug delivery. Finally, we will present key challenges and novel approaches that we can utilize in the future in order to effectively study MSC/BBB interactions *in vivo* and develop MSC-based therapeutics to treat neurological diseases. The study of exogenous MSC homing and distribution into the CNS will not only shed light on how transplanted MSCs exert their therapeutic functions but also will allow us to gain insight into how endogenous MSCs migrate, traffic, and function in response to either CNS injury or other diseases. Additionally, studying MSC trafficking across the BBB may also contribute to the development of methods to monitor the fate of endogenous and exogenous stem cells *in vivo*.

## 2. Leukocyte Transmigration across the Blood-Brain Barrier (BBB)

The BBB is formed by cellular interactions between brain microvascular endothelial cells (BMECs), astrocytes, pericytes, and neurons [11, 12]. CNS endothelial cells (ECs) exhibit three characteristics that establish their BBB properties. (a) ECs have TJs that restrict diffusion of ions and polar molecules, resulting in high electrical resistance (TEER) [13, 14]. Endothelial TJs in the CNS are composed of transmembrane proteins Claudins (-5, -12), Occludin, and junctional adhesion molecules (JAMs), as well as cytoplasmic anchoring proteins such as Zonula Occludens proteins (ZO-1, ZO-2). These proteins regulate the paracellular (i.e., between ECs) permeability of endothelial cells [13, 15]. (b) CNS but not peripheral ECs contain a small number of endocytotic caveolae that serve as intermediates in the receptor-dependent and -independent transcytosis [16]. Caveolae are characterized by expression of Caveolins (Cav-1, -2, and -3), a class of transmembrane proteins (21–24 kDa) that are essential for caveolae formation [17, 18]. Notably, expression of Cav-1 is upregulated prior to BBB breakdown following CNS injury or stroke, concurrent with the increased rate of transcytosis [19, 20]. (c) Finally, CNS endothelium expresses a large number of specific active or passive transporters that regulate passage of nutrients (e.g., glucose or amino acids) from the blood to the brain and prevent drug delivery [15, 21]. 

**Figure 1:**
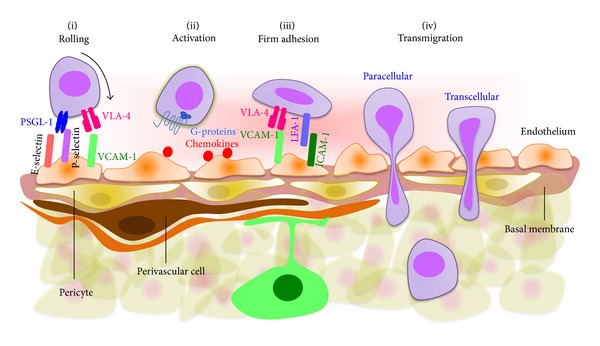
Leukocyte extravasation cascade. Leukocytes initially engage with the endothelium via selectin and VCAM-1 mediating interactions during rolling (i), followed by G-protein-mediated activation (ii) and subsequent integrin-mediated firm adhesion (iii). Transmigration across the BBB may occur via paracellular or transcellular routes (iv). It remains to be determined whether systemically infused MSCs possess similar or distinct features and mechanisms enabling them to transmigrate across the BBB and home to the CNS system *in vivo*.

The BBB plays a vital role in brain homeostasis by restricting the passage of molecules and leukocytes into and out of the brain [22]. However, during brain inflammation and injury, the BBB becomes compromised and cellular trafficking through the BBB is significantly upregulated [23]. Leukocyte trafficking to sites of CNS inflammation has been well studied and extensively reviewed [22, 24]. We will only provide a brief overview in order to contrast leukocyte and MSC transmigration across the BBB. Circulating leukocyte transmigration (also called extravasation or diapedesis) through the BBB occurs primarily at postcapillary venules and is characterized by a multistep adhesion/migration cascade (Figure 1) [25, 26]. During inflammation, BMECs upregulate cell surface adhesion molecules (e.g., P- and E-selectins, vascular cell adhesion molecule-1 (VCAM-1) and Intercellular Adhesion Molecule-1 (ICAM-1)), and chemoattractants (e.g., stromal cell-derived factor-1 (SDF-1) (or CXCL12) and CCL19). Leukocytes initiate transient selectin-mediated tethering and rolling on the endothelium that triggers activation of leukocyte integrins such as leukocyte function-associated molecule-1 (LFA-1, ligand for ICAM-1), macrophage-1 antigen (Mac-1, ligand for ICAM-1), and very late antigen-4 (VLA-4, ligand for VCAM-1) and leads to leukocyte arrest on ECs. Leukocytes then undergo actin-dependent polarization and Mac-1/ICAM-1-mediated lateral “crawling” over the luminal surface. Eventually, leukocytes migrate across the endothelial barrier through both paracellular (i.e., between endothelial cells (ECs)) and transcellular (i.e., directly through individual ECs) pathways, although the transcellular route is preferred [27, 28]. Adhesion of leukocytes on the EC layer induces clustering of endothelial cell surface adhesion molecules (i.e., ICAM-1 and VCAM-1) and triggers downstream signaling pathways that disrupt junctions and promote paracellular migration. Conversely, during transcellular migration, interactions between ICAM-1 and VCAM-1 on the EC surface induce formation of vertical microvilli-like projections (called “transmigratory cups”) [27] that provide directional guidance for leukocyte extravasation. Transcellular migration seems to play a major role in leukocyte trafficking in the CNS system where ECs have strong tight junctions [29]. Actin-containing protrusive structures (e.g., podosomes, filopodia, lamellipodia, and pseudopodia) are often formed in leukocytes to enable them to “probe” into, and subsequently penetrate, ECs [27]. In contrast, in some types of CNS injury, activation of ECs and astrocytes can lead to reduced TJ integrity and formation of paracellular gaps, thereby facilitating the migration of leukocytes through a paracellular route. After passing through the endothelial barrier, leukocytes can then penetrate the endothelial basement membrane (BM) and pericytes through gaps within the ECM facilitated by matrix metalloproteinase- (MMP-)mediated ECM degradation. 

## 3. Tropism of MSCs towards Brain

MSCs delivered systemically have been shown to preferentially localize to sites of inflammation, injury, ischemic lesions, and tumors including those in the brain despite their predominant entrapment in the lung vasculature [3, 30, 31]. For instance, Yilmaz et al. found that intravenously (IV) injected mouse bone-marrow-derived MSCs home to the infarct site in the transient middle cerebral artery occlusion (t-MCAO) model for stroke [31]. The brain tropism for MSCs was confirmed by whole body imaging of radiolabeled MSC given to rats with and without t-MCAO. During the first two hours after stroke, MSCs are transiently trapped in the lungs but migrate over time within the region of brain ischemia [32]. Kim et al. also found that human adipose-derived MSCs (hAMSCs) transplanted through an i.v. route crossed the BBB and migrated into the brain in a mouse model for AD [32, 33]. Systemically infused MSCs can also selectively accumulate into certain brain tumors (e.g., gliomas) [8, 10, 34–36]. These studies suggest that MSCs may possess leukocyte-like, active homing mechanisms that enable them to interact with and migrate across the BBB under injury or inflammation. However, the integrity of the cerebral vasculature is likely compromised following injury or inflammation, which can lead to passive MSC accumulation in the brain via entrapment [37]. Therefore, the extent and mechanisms of how MSCs actively cross the BBB remain to be determined. 

## 4. Molecular Mechanisms of MSC/BBB Interaction and Transmigration

Several studies have shown that MSCs can utilize a leukocyte-like, multistep homing cascade (i.e., rolling, adhesion, and transmigration) to engage with ECs. However, a major caveat of the studies that we will discuss below is the use of cultured EC monolayers including non-BMECs such as human umbilical vein ECs (HUVECs) that do not fully acquire BBB properties typical of the *in vivo* situation.

MSCs express a variety of leukocyte homing molecules such as chemokine receptors (e.g., CXCR4, CCR2) and cell adhesion molecules (e.g., CD44, integrins *α*4 and *β*1, and CD99), while they lack some key homing markers including P-selectin glycoprotein ligand 1 (PSGL-1), LFA-1, and Mac-1 [38]. However, studies of MSC-EC interactions and subsequent transmigration have produced conflicting results. Rüster et al. reported that MSCs interact with activated ECs under flow conditions via P-selectin during the initial tethering and rolling steps, although MSCs do not express common P-selectin ligands such as PSGL-1 and CD24 [39]. However, they found that the rolling velocity of MSCs on HUVEC is 100–600 *μ*m/s under shear stress of 0.1–1 dyn/cm^2^, a value that is significantly higher than that of leukocytes (~2–100 *μ*m/sec under physiologically relevant shear stress of 1–4 dyn/cm^2^) [39]. On the contrary, several studies reported that MSCs are not able to interact with ECs under flow conditions [40, 41]. Sackstein et al. showed that native MSCs do not express either PSGL-1 or major functional moieties involved in cell rolling such as sialyl Lewis X (SLeX) and therefore do not bind to P- and E-selectins. MSCs therefore have minimal binding interactions with ECs and they only modestly infiltrate the bone marrow [40]. Similar results were also obtained by Brooke and coworkers [42]. Furthermore, the role of VCAM-1/VLA-4, a receptor/ligand pair that mediates both cell rolling and adhesion, in MSC homing is unclear; few reports [43–45] including that of Rüster et al.'s [39] stated that VCAM-1/VLA-4 interactions are involved in MSC firm adhesion on ECs and transmigration while others found that MSCs do not bind to VCAM-1 [46]. 

Several studies also investigated MSC transmigration through *in vitro* endothelial monolayers [40, 45, 47]. In a coculture system of MSC with an endothelial cell monolayer, Steingen and coworkers found that MSCs transmigrated through the endothelial barrier using adhesion molecules including VCAM-1/VLA-4 and *β*1 integrin [45]. When MSCs were perfused into an isolated heart and investigated using electron microscopy, the authors observed that the tight junctions between endothelial cells became abolished and MSCs interacted with the endothelial cell layer in association with tight cell-cell contacts. In a recent work published by Teo and coworkers, high-resolution confocal and dynamic live-cell imaging has supported an active mode of MSC transmigration across various EC monolayers from lung microvascular endothelial cells (LMVECs) to rat brain ECs (GPNT, a cell line previously used to model the BBB *in vitro*) [48]. MSCs preferentially transmigrate on TNF*α*-activated endothelium, rather than naïve endothelium, using VCAM-1 and G-protein-coupled receptor signaling- (GPCR-)dependent pathways. MSCs migrate either by paracellular or transcellular diapedesis through discrete gaps or pores in the endothelial monolayer that are enriched for VCAM-1 (transmigratory cups). In contrast to leukocytes, MSC transmigration does not involve significant lateral crawling, presumably due to the lack of Mac-1 expression. Interestingly, MSC exhibited nonapoptotic membrane blebbing activity in the early stages of endothelial transmigration rather than formation of lamellipodia and invadosomes that are normally found in leukocytes, to potentially breach endothelial cells. Finally, MSC transmigration occurred on the time scale of hours. Although the mechanism of MSC transmigration is comparable to leukocyte transmigration across the BBB in some studies, the time is much longer than leukocyte transmigration in other endothelial systems (usually within minutes) [48]. Yilmaz et al. have studied trafficking of IV-injected mouse bone-marrow-derived MSCs to the brain in the t-MCAO model *in vivo* and found that interactions between the CD44 on MSCs and P- and E-selectins on ECs mediate MSC recruitment to the CNS [31]. Matsushita et al. have also found that rat MSCs could migrate through a monolayer of rat BMECs *in vitro* via a paracellular pathway [47] although the underlying mechanism was not reported. Furthermore, Lin et al. recently reported that MSCs trigger tight junction disassembly in human BMEC monolayers through PI3K and ROCK signaling pathways [49]. 

Similar to immune cells, chemokine receptors and their chemokine ligands are also found to be involved in MSC migration and endothelial transmigration [50–53]. For instance, Chamberlain et al. demonstrated functional expression of various chemokine receptors on murine MSCs using standard Boyden-type chamber assays [50]. More recently, they found that CXCL9, CXCL16, CCL20, and CCL25 were specifically involved in MSC transendothelial migration across murine aortic endothelial cells (MAECs) [41]. In Bloch's studies, they found that cocultivation of MSCs in the presence of bFGF, VEGF, EPO, and IL-6 resulted in a significant increase of MSC integration with the EC monolayer. They also found that VEGF, EPO, and IL-6 enhanced transmigration, although to different extents, whereas bFGF significantly decreased the transmigration of MSCs [45]. Furthermore, Feng et al. demonstrated that interactions of chemokines and chemokine receptors, specifically through fractalkine-CX3CR1 and SDF-1-CXCR4, partly mediated the migration of rat MSCs to the impaired site in the brain after hypoglossal nerve injury [54].

Finally, the activation of MMPs is also found to be associated with MSC transendothelial migration via degradation of the endothelial BM *in vitro*, providing a potential mechanism for MSC homing and extravasation into injured tissues *in vivo* [55]. MSCs constitutively express MMP-2 and membrane type 1 MMP (MT1-MMP) that may play a role in MSC invasion in reconstituted BM matrigel. In particular, Becker et al. [55] found that MSC transmigration across *in vitro* bone marrow endothelium is at least partially regulated by MMP-2. Interestingly, they also demonstrated that high culture confluence of MSCs was found to increase production of the endogenous MMP-inhibitor TIMP-3 and decrease transendothelial migration of MSCs. The involvement of MMPs in MSC transmigration is also supported by Bloch's study where MSCs-derived MMP-2 but not MMP-9 is found at sites of BM invasion and degradation [45]. Interestingly, TIMP3 expressed by IV administered MSCs is a key player in ameliorating BBB permeability in rodent models after traumatic brain injury (TBI) by blocking vascular endothelial growth factor-A-induced breakdown of endothelial cell adherens junctions [56]. These findings elucidate a potential molecular mechanism for the beneficial effects of MSCs in treating neurological diseases through regulation of BBB integrity.

## 5. MSC as a Delivery Vehicle for Brain Tumors

The fact that the BBB restricts the passage of molecules of molecular weight >400 Dalton presents a great challenge in delivering therapeutics to treat brain tumors and certain CNS diseases. Besides their endogenous therapeutic effects, the tropic properties of MSCs provide unique opportunities to use them as vehicles for gene and drug delivery to treat brain tumors. For instance, Nakamizo and coworkers have demonstrated that MSCs were capable of migrating into glioma xenografts *in vivo* after intravascular or local delivery [35]. They also found that MSCs engineered to produce IFN-*β* significantly increased animal survival compared with controls in a U87 intracranial glioma xenograft mouse model [35]. Recently, Kim et al. have tested combination therapy for malignant glioma with TRAIL-secreting MSCs and the lipoxygenase inhibitor MK886 that can increase sensitivity to TRAIL-induced apoptosis [8]. They found that MSC-based TRAIL gene delivery combined with MK886 had greater therapeutic efficacy than single-agent treatment in an orthotopic glioma xenografted mouse model [8]. Interestingly, MSCs can also be used as target-delivery vehicle for anticancer drug-loaded biodegradable nanoparticles [57]. This approach may be advantageous over genetic modification with respect to safety and controlled drug release. Roger et al. have found that coumarin-6 containing polylactic acid nanoparticles and lipid nanocapsules can be efficiently internalized into MSCs without affecting cell viability or differentiation [36]. Furthermore, they reported that nanoparticle-loaded cells were able to migrate toward an experimental human glioma model, suggesting that MSCs can serve as cellular carriers for drug-loaded nanoparticles to treat brain tumors. 

## 6. Conclusion and Perspectives

MSC transplantation via systemic administration holds enormous potential to treat numerous neurological and brain diseases. However, the *in vivo* efficacy of MSC therapy has not been well established, and some recent clinical trials have produced mixed results [2, 3]. The lack of efficacy is attributed largely to an incomplete understanding of MSC biology and their fate following transplantation *in vivo* [2, 3]. In particular, crossing the BBB may be a prerequisite for MSCs to exert their therapeutic effects in treating neurological diseases or CNS injury [3, 30, 31] and is necessary for their use as vehicles for drug delivery to treat brain tumors [58]. It seems clear that, at least *in vitro*, MSCs possess leukocyte-like, although inefficient, molecular mechanisms involving adhesion molecules, chemokines, and proteases which enable MSC/EC interactions and transmigration. The large discrepancies between studies may be due to the inherent heterogeneity of MSCs combined with variations in experimental techniques and models. A major caveat of *in vitro* studies is the use of EC monolayers that do not fully recapitulate the *in vivo* BBB properties. It will be important to incorporate other BBB cell types, such as primary astrocytes, pericytes, reconstituted basement membrane, and relevant dynamic flow conditions in order to develop more robust *in vitro* systems for studying MSC/EC interactions. Despite the *in vitro* evidence, it remains elusive whether systemically infused MSCs are able to use leukocyte-like homing cascades to actively interact with and transmigrate across the BBB *in vivo* under both normal and pathological conditions. Indeed, it is not clear if MSCs are actually able to actively home or rather are passively “captured” at sites of inflamed and disrupted vessels. Physical factors may act in concert with active homing mechanisms to stop or slow down cells before adhesion interactions subsequently arrest MSCs on ECs. 

In order to fully understand the dynamic behavior of transplanted MSCs, imaging of transplanted cells in both the brain and other tissues is required. Both short- and long-term monitoring of cell fate *in vivo* have benefited from improved molecular imaging techniques to visualize cell survival, biodistribution, and behavior [59–62]. Magnetic resonance-based tracking of transplanted cells has confirmed that MSCs rapidly localize to infracted regions of the brain [63–65]. Alternatively, a powerful approach for understanding transplanted cell behavior at the single-cell level is to utilize intravital imaging techniques to study MSC/BBB interactions. In particular, novel transgenic models where TJs between endothelial cells of the BBB or endothelial caveolae are fluorescently tagged may illuminate the mode and dynamics of MSC transmigration in the brain and elsewhere [59]. The study of exogenous MSC homing mechanisms *in vivo* will not only shed light on how transplanted MSCs exert their therapeutic functions in treating neurological diseases but also will allow us to gain insight into how endogenous MSCs migrate, traffic, and function in response to injury. The mechanistic study of MSC tropism to the brain will also facilitate development of MSCs that are engineered with key homing molecules through genetic or chemical modifications in order to improve MSC targeting and drug delivery in case their basal homing process is inefficient [40, 60]. Finally, the elucidation of stem cell fate following transplantation that is believed to be a major bottleneck in stem cell therapy will have broad implications in understanding stem cell functions and developing more effective stem-cell-based therapeutics [2, 3].
